# Precision Medicine in Radiomics and Radiogenomics

**DOI:** 10.3390/jpm12111806

**Published:** 2022-11-01

**Authors:** Serena Monti

**Affiliations:** Institute of Biostructures and Bioimaging, National Research Council, 80145 Napoli, Italy; serena.monti@ibb.cnr.it

Precision medicine is an innovative and emerging approach to treatment that accounts for individual variability in genetic and environmental factors to identify and utilize the specific biomedical profile of a patient’s disease. It addresses the common observation that patients with apparently the same clinical diagnosis or symptoms often exhibit different responses to the same treatment. It is also sometimes known as “personalized medicine”; however, the National Academy of Sciences of the USA does not prefer this definition, because this approach does not literally mean the creation of drugs or medical devices that are unique to a patient, but rather the ability to classify individuals into groups differing in their susceptibility, prognosis, or response to a particular disease and treatment [1]. 

Several definitions exist for precision medicine, but one of the most effective is the one given by the European Society of Radiology, which states that the goal of precision medicine is to target the right treatments for the right patients at the right time. This translates to better diagnoses, prompt interventions, more effective treatments with reduced side effects and improved cost-effectiveness [2]. All these promises pass through the identification of patient features called biomarkers, which are biological characteristics that are objectively measured and evaluated as indicators of biological processes [3]. Consequently, advances in genomics, molecular biology, information technology, and imaging are the key drivers in accelerating both the achievements and the acceptance of precision medicine, which has been receiving growing recognition by clinicians, healthcare systems, pharmaceutical companies, patients, and the government in recent years.

In this context, imaging plays an essential role because it allows screening, early diagnosis, response evaluation, and recurrence assessment in a noninvasive way [4]. In addition, the integration of multiparametric [5,6] (structural, functional, molecular, and metabolic) information provided by imaging techniques has greatly increased the ability of clinicians and researchers to group patient populations into phenotypic subsets that share similar prognoses and are likely to respond to similar therapies.

A field that shows great promise in this context is radiomics, a new ”data-driven” approach to the analysis of medical images. Radiomics is defined as “the high-throughput mining of quantitative image features from standard-of-care medical imaging that enables data to be extracted and applied within clinical-decision support systems to improve diagnostic, prognostic, and predictive accuracy” [7]. Radiomics aims to identify and combine new imaging biomarkers, invisible to the human eye, in a mathematical model, providing predictive or prognostic information about patients and their pathologies based on sophisticated statistical analyses. These biomarkers are called radiomic features, i.e., image descriptors reflecting tissue heterogeneity and, indirectly, molecular and genetic substrates. Radiomic features should be extracted through a high-quality pipeline in order to be reproducible and quantitative. They then undergo a selection process and are combined using a classifier (support vector machine, random forest, etc.).

The further correlation of imaging phenotypes with gene expressions is known as radiogenomics. In radiation therapy, this term refers to the study of the correlation between genetic variation and response to radiation therapy [8]; in the radiology community, radiogenomic studies aim to determine statistically significant linkages between imaging features and gene expressions, creating models that predict patient outcomes based on imaging features. It will serve as the foundation for the surveillance of disease manifestation in terms of occurrence, location, extent, severity, and discovery of genetic polymorphisms. The concept behind radiogenomics is the possibility of investigating the relationship between imaging, genomics, and clinical knowledge simply by looking at data; therefore, radiogenomic approaches, such as radiomics, are based on numerical calculus and computer science methods, allowing the management and analysis of a very large number of variables [9].

The research community and funding agencies have been somewhat slow to recognize the value of collecting imaging data in conjunction with genomic data. For example, while The Cancer Genome Atlas, collecting clinical and genomic data, was initiated by the National Institutes of Health in 2006 [10], the Cancer Imaging Archive, providing the corresponding clinical images, was created much later [11]. However, in recent years, the interest of the scientific community in radiomics and radiogenomics has exponentially increased. A simple search on Scopus^®^ (TITLE-ABS-KEY (radiomics) OR TITLE-ABS-KEY (radiogenomics)) shows this growing trend, with a number of manuscripts that, starting from less than ten articles per year before 2012, reached 2390 articles in the last year. 

Oncology is at the forefront of the development of radiomic and radiogenomic applications. For example, in the field of breast cancer, distinct histological subtypes and at least four different molecular subtypes have been established. Molecular breast cancer subphenotypes are responsible for disease expression, response to treatment, and patient survival outcomes. Genetic analysis has been replaced by immunohistochemical surrogates of molecular subtypes; however, the establishment of relationships between tumor genomic characteristics and their imaging phenotypes can provide clinically relevant prognostic information, because each breast cancer subtype is associated with a unique prognosis [12]. Indeed, The Cancer Genome Atlas Breast Phenotype Research Group has undertaken major research initiatives in breast cancer, and have reported significant correlations between imaging phenotypes and breast cancer phenotypes [13]. However, similar advantages have been obtained in the brain tumor research field, where researchers are integrating the knowledge of tumor molecular characteristics into molecular imaging techniques to use noninvasive imaging modalities which can classify patients into subgroups with similar tumor characteristics and prognoses that may benefit from similar treatment strategies. Another clear example of the increasing role of imaging in precision medicine is prostate cancer [14,15]: multiparametric MRI is a potent tool for diagnosing prostate cancer and for classifying patients into subgroups with different risk levels, which helps in planning treatment and predicting prognoses and oncological outcomes. 

While radiomics and radiogenomics applications are fewer among noncancerous conditions, several advancements have been made in defining imaging phenotypes, particularly in fields in which reproducible quantitative imaging biomarkers have been validated and used to clinically manage patients, e.g., neurodegenerative and neuroinflammatory diseases [16,17,18] or chronic liver diseases [19,20].

Despite the dramatic increase in the number of published studies, it is important to outline acknowledge the challenges associated with these new approaches. Often, radiomic and radiogenomics studies have reported correlations which do not necessarily imply causation between imaging biomarkers and patient outcomes. In order to move forward in this field, it is necessary to conceive experiments with standardized acquisition and processing techniques and to validate existing radiomic and radiogenomics models. 

Radiomics and radiogenomics have proven their potential, despite not yet being integrated in routine clinical practice. However, these new perspectives may deeply change the paradigm of clinical routines in the near future, assigning a prominent role to imaging in the management of complex, genetically heterogeneous diseases of both oncological and non-oncological conditions.

## Data Availability

Not applicable.
